# Clinical Risk Stratification Using the STUMBL (STUdy of the Management of BLunt Chest Wall Trauma) Score in Blunt Chest Trauma: A Prospective Observational Study

**DOI:** 10.7759/cureus.104517

**Published:** 2026-03-01

**Authors:** Dilpreet Singh Dhillon, Nishith S Mandal, Aakansha Giri Goswami, Jatin Chavda, Pramatheshwara S Aradhya, Sneha S Chittari, Ravdeep Kaur, Shourya Vijayvargia

**Affiliations:** 1 General Surgery, Vardhman Mahavir Medical College and Safdarjung Hospital, New Delhi, IND; 2 Intern, Atal Bihari Vajpayee Institute of Medical Sciences and Dr. Ram Manohar Lohia Hospital, New Delhi, IND

**Keywords:** blunt chest trauma, clavien-dindo classification, complication severity, rib fractures, risk stratification, stumbl score, trauma outcomes

## Abstract

Background: Blunt chest trauma is a common cause of emergency department presentation. It is associated with a broad spectrum of clinical outcomes, ranging from minor chest wall injuries to life-threatening respiratory complications. Early risk stratification remains challenging, and reliance on clinical judgment alone may fail to identify patients at risk of deterioration. The STUMBL (STUdy of the Management of BLunt chest wall trauma) score was developed as a bedside tool to stratify risk in such patients; however, data correlating STUMBL score strata with graded complication severity remain limited.

Methods: This prospective observational cohort study was conducted at a tertiary care teaching hospital in India and included consecutive patients presenting with blunt chest trauma to the emergency department over 18 months. STUMBL scores were calculated on presentation. Patients were followed throughout hospitalization to assess clinical outcomes, including complications, intensive care unit (ICU) admission, length of hospital stay, and in-hospital mortality. Complication severity was graded using the Clavien-Dindo classification. The primary outcome was in-hospital complication severity graded using the Clavien-Dindo classification on day 3 of admission.

Results: Ninety patients were included in the analysis. The mean STUMBL score was 5.6 ± 6.7 (range: 1-39). A significant association was observed between STUMBL score categories and Clavien-Dindo complication severity on day 3 (χ² = 65.2, p < 0.001). Patients with STUMBL scores ≥ 16 demonstrated a marked escalation in complication severity and resource utilization.

Conclusions: The higher STUMBL score categories were significantly associated with increased in-hospital complication severity and greater healthcare resource utilization. The STUMBL score may serve as a useful clinical risk stratification tool in patients with blunt chest trauma. Further multicenter studies with larger sample sizes are warranted to validate these findings.

## Introduction

Blunt chest trauma is a frequent cause of emergency department presentation and contributes substantially to trauma-related morbidity and mortality worldwide. Mechanisms such as road traffic collisions, falls, and physical assaults commonly result in chest wall injuries, including rib fractures, pulmonary contusions, pneumothorax, and hemothorax. While some patients experience an uncomplicated clinical course, others develop significant respiratory compromise, pneumonia, or require critical care support, underscoring the heterogeneity of outcomes associated with blunt thoracic injury [1].

Accurate early risk stratification is central to effective management of blunt chest trauma. Traditional assessment relies heavily on clinical judgment, supported by physiological parameters and imaging findings. However, early clinical presentation may underestimate injury severity, particularly in patients who initially appear stable but later deteriorate. This has prompted increasing interest in structured clinical scoring systems designed to identify patients at higher risk of adverse outcomes at an early stage [2].

Several trauma scoring systems, including the injury severity score (ISS) and trauma and injury severity score (TRISS), have been used to assess overall injury burden but are not specific to thoracic trauma and may lack sensitivity for predicting chest-specific complications [3,4]. More focused tools, such as the rib fracture score and RibScore, incorporate anatomical injury patterns but do not consistently account for physiological reserve or comorbid factors known to influence outcomes [5]. The STUMBL (STUdy of the Management of BLunt chest wall trauma) score was developed as a pragmatic bedside tool incorporating age, oxygen saturation, number of rib fractures, pre-existing lung disease, and prior anticoagulant use. Studies have demonstrated its utility in estimating the risk of complications and the need for higher level care in patients with blunt chest trauma [6-8]. However, most published work has focused on binary outcomes such as complication presence or intensive care unit (ICU) admission.

Standardized assessment of complication severity may offer additional clinical insight beyond simple outcome occurrence. The Clavien-Dindo classification provides a validated, reproducible framework for grading complications based on the level of intervention required and has been increasingly applied beyond postoperative settings to trauma populations [9,10]. Data correlating STUMBL score strata with graded complication severity using such standardized classifications remains limited.

Although the STUMBL score has been evaluated in relation to complication occurrence and need for intervention, prior studies have primarily focused on binary outcomes or predictive validation metrics. The relationship between STUMBL score categories and standardized complication severity grading has not been prospectively examined. In particular, graded escalation of complication severity using structured frameworks such as the Clavien-Dindo classification has not been systematically correlated with STUMBL score strata in real-world trauma cohorts. This study was therefore undertaken to evaluate the association between STUMBL score categories and complication severity as assessed by the Clavien-Dindo classification in patients with blunt chest trauma.

## Materials and methods

Study design and setting

This was a prospective observational cohort study conducted over 18 months (April 2023 to September 2024) in the emergency department and surgical services of Vardhman Mahavir Medical College and Safdarjung Hospital, New Delhi, India, a tertiary care teaching hospital. Consecutive sampling was used, and all eligible patients presenting with blunt chest trauma during the study period were screened for inclusion and enrolled prospectively.

Ethical approval

The study protocol was reviewed and approved by the Institutional Ethics Committee before study initiation. Written informed consent was obtained from all participants or their legally authorized representatives in accordance with institutional guidelines and the Declaration of Helsinki.

Study population

The study included patients aged over 12 years who presented to the emergency department with blunt chest trauma. Patients were excluded if they had a Glasgow coma scale score of less than 15 on presentation, sustained penetrating chest injuries, or were unwilling or unable to provide informed consent.

Sample size calculation

The sample size was calculated using the formula for estimating a single population proportion:

\[n = \frac{Z^2 \times p \times (1 - p)}{d^2}\]

where n is the required sample size; Z is the standard normal deviate corresponding to a 95% confidence level (1.96); p is the anticipated proportion of complications; and d is the absolute precision.

Based on prior literature, an anticipated complication prevalence of 16.8% in patients with blunt chest trauma and a reported specificity of 77.9% for the STUMBL score in identifying complications were used to inform the expected event rate [11]. Using a two-sided alpha error of 5% and an absolute precision of 10%, the minimum required sample size was calculated as 79 patients. To account for potential exclusions and incomplete data, the final target sample size was rounded to 90 patients.

The sample size was calculated to estimate the anticipated prevalence of in-hospital complications in blunt chest trauma with acceptable precision. The study was not specifically powered for subgroup comparisons across STUMBL score categories.

Clinical assessment and management

All enrolled patients were assessed and managed according to advanced trauma life support (ATLS) principles on presentation [12]. Initial stabilization included airway assessment, hemodynamic resuscitation, and oxygen supplementation as required. Baseline clinical parameters, including heart rate, blood pressure, oxygen saturation on room air, and time from injury to presentation, were recorded.

Radiological evaluation

All patients underwent initial chest radiography as part of routine trauma evaluation. An extended focused assessment with sonography for trauma (eFAST) was performed in all cases to assess for thoracic complications [13]. Computed tomography (CT) of the chest was obtained selectively at the discretion of the treating team based on clinical and radiological findings.

STUMBL score assessment

The STUMBL score was calculated at presentation for each patient using predefined variables, including age, oxygen saturation on room air, number of rib fractures identified on imaging, history of pre-existing lung disease, and use of anticoagulant therapy before injury. Each variable was scored according to the original STUMBL scoring system, and the total score was calculated by summation [14]. Patients were subsequently categorized into predefined STUMBL score ranges to assess trends across increasing score strata.

Outcome measures

The primary outcome was the occurrence and severity of in-hospital complications, graded using the Clavien-Dindo classification on day 3 of admission [9]. Secondary outcomes included ICU admission, length of hospital stay, day 30 complication status, and in-hospital mortality. Pneumonia was defined based on the presence of new or progressive infiltrates on chest imaging accompanied by compatible clinical features (fever, leukocytosis, or purulent sputum). Patients were assessed daily during hospitalization for the development of pulmonary complications.

The secondary outcomes were the association between STUMBL score categories and radiological findings on chest X-ray and CT, as well as trends in healthcare resource utilization across increasing STUMBL score categories, including ICU admissions and hospitalization duration.

Follow-up

Patients were followed throughout their hospital stay. Complication severity was assessed on day 3 and day 30 using the Clavien-Dindo classification to capture both early and short-term outcomes.

Statistical analysis

Data were analyzed using IBM SPSS Statistics for Windows, version 21.0 (IBM Corp., Armonk, NY). Statistical analyses were performed using appropriate categorical methods. Associations between STUMBL score categories and clinical outcomes were assessed using chi-square tests or Fisher’s exact tests where expected cell counts were < 5. Continuous variables were summarized using mean ± standard deviation and compared descriptively across STUMBL score categories.

Given the exploratory design, modest sample size, and limited number of severe outcome events in higher STUMBL strata, multivariable regression, correlation-based modeling, and ROC analysis were not performed. The study was designed to evaluate graded associations for clinical risk stratification rather than predictive modeling.

## Results

Study population

A total of 90 consecutive patients with blunt chest trauma were included in the final analysis. No missing data was recorded for the primary outcome variables.

Baseline demographic and clinical characteristics

The mean age of the cohort was 32.0 ± 13.9 years (range: 16-82 years). Most patients were male (86%, n = 77). The mean time from injury to hospital presentation was 9.8 ± 5.4 hours (range: 2-36 hours). Falls were the most common mechanism of injury (61%, n = 55), followed by road traffic incidents (21%, n = 19) and physical assault (18%, n = 16). Baseline demographic and clinical characteristics of the study population are summarized in Table 1.

**Table 1:** Baseline demographic and clinical characteristics of the study population (N = 90) MAP: Mean arterial pressure.

S. No	Characteristics	Values
1	Age, mean ± SD (years)	32.0 ± 13.9
2	Male sex, n (%)	77 (86%)
3	Time to presentation, mean ± SD (hours)	9.8 ± 5.4
4a	Mechanism of injury – falls, n (%)	55 (61%)
4b	Mechanism of injury – road traffic incidents, n (%)	19 (21%)
4c	Mechanism of injury – physical assault, n (%)	16 (18%)
5	Tachycardia on presentation, n (%)	66 (73%)
6	Hypotension (MAP < 65 mmHg), n (%)	2 (2.2%)
7	Oxygen saturation on room air, mean ± SD (%)	98.1 ± 1.2

Hemodynamic and physiological parameters at presentation

On presentation, 73% (n = 66) of patients were tachycardic (pulse rate above 100 beats per minute). Only 2.2% (n = 2) were in shock (mean arterial pressure < 65 mmHg). Mean oxygen saturation on room air was 98.1 ± 1.2% (range: 92-100), with hypoxemia (SpO₂ < 95%) observed in 2.2% (n = 2) of patients.

These findings suggest that most patients appeared physiologically stable at initial assessment despite subsequent complications in higher STUMBL score categories.

Radiological findings

Rib fractures were identified on chest radiography in 21.1% of patients (n = 19). Among these patients, 8.9% (n = 8) developed hemothorax, and 4.4% (n = 4) developed pneumothorax. Extended focused assessment with sonography for trauma (eFAST) was positive in 13.2% of patients (n = 12). Chest CT was performed selectively in 5.5% of patients (n = 5) with concerning clinical or radiographic features, demonstrating pulmonary contusions in four patients and a pulmonary contusion with atelectasis in one patient (Table 2).

**Table 2:** Radiological findings in the study population eFAST: Extended focused assessment with sonography for trauma.

Radiological findings	Patients, n (%)
Rib fractures on chest radiography	19 (21.1%)
Hemothorax	8 (8.9%)
Pneumothorax	4 (4.4%)
Positive eFAST	12 (13.2%)
Chest CT performed	5 (5.5%)
Pulmonary contusion on CT	4 (4.4%)
Pulmonary contusion with atelectasis on CT	1 (1.1%)

A statistically significant association was observed between STUMBL score categories and the presence of rib fractures (chi-square test, χ² = 28.92, p = 0.00002). Pneumothorax was also significantly associated with higher STUMBL score categories (χ² = 39.27, p = 0.0000002), as was hemothorax, which demonstrated the strongest association with high STUMBL score range (χ² = 48.16, p = 0.000000003). Rib fractures, pulmonary contusions, hemothorax, and pneumothorax were more frequently observed in higher STUMBL score categories. Pulmonary contusions and atelectasis were observed almost exclusively in patients with STUMBL scores ≥ 16, indicating an increasing radiological injury burden with escalating STUMBL score categories.

Distribution of complications across STUMBL score categories

The mean STUMBL score was 5.6 ± 6.7 (range: 1-39). The contribution of individual STUMBL score components is summarized in Table 3.

**Table 3:** Distribution of individual STUMBL score components in the study cohort (n = 90) STUMBL: STUdy of the Management of BLunt chest wall trauma.

STUMBL score component	Mean ± SD	Observed range
Pre-existing lung disease	0.2 ± 0.9	0–5
Pre-injury anticoagulant use	0.1 ± 0.5	0–4
Number of rib fractures	2.5 ± 6.5	0–36
Oxygen saturation (SpO₂)	0.04 ± 0.3	0–2
Age	2.7 ± 1.5	1–8

Patients were stratified into six predefined STUMBL score categories (0-10, 11-15, 16-20, 21-25, 26-30, and ≥31). The majority of patients (90%, n = 81) were in the lowest score category (0-10). The number of patients decreased progressively across higher score strata, with only one to two patients in each of the highest categories.

The distribution of observed thoracic complications across STUMBL score categories is shown in Table 4. Pulmonary contusions were not observed in patients with STUMBL scores ≤ 15 and first appeared in the 16-20 category. Their occurrence was confined to patients with STUMBL scores ≥ 16. Atelectasis was rare, with a single case recorded in the highest STUMBL score category (≥31). Hemothorax was observed across multiple STUMBL score ranges, including the lowest category (0-10), but was numerically more frequent in higher score strata. Pneumothorax was observed in the lower STUMBL categories (0-10 and 11-15), absent in the intermediate categories (21-25 and 26-30), and present again in the 16-20 range. No cases of pneumonia were recorded in the study cohort. Overall, higher STUMBL score categories were associated with a greater burden of thoracic complications; however, interpretation of trends in the higher score ranges is limited by the small number of patients within those strata.

**Table 4:** Observed thoracic complications across STUMBL score categories * Estimated risk derived from the original STUMBL score description [14]. STUMBL: STUdy of the Management of BLunt chest wall trauma.

STUMBL score category	Patients, n (%)	STUMBL-estimated risk of complications*	Pulmonary contusion	Atelectasis	Hemothorax	Pneumothorax
0–10	81 (90.0%)	13%	0	0	3	1
11–15	3 (3.3%)	29%	0	0	0	2
16–20	2 (2.2%)	52%	1	0	1	1
21–25	1 (1.1%)	70%	1	0	1	0
26–30	1 (1.1%)	80%	1	0	1	0
≥31	2 (2.2%)	88%	2	1	2	0

STUMBL score and in-hospital complications

Increasing STUMBL score categories were associated with higher frequencies of thoracic complications, including pulmonary contusion, hemothorax, pneumothorax, and atelectasis. Pulmonary contusions and atelectasis were observed almost exclusively in patients with STUMBL scores ≥16. No cases of pneumonia were recorded during the study period.

STUMBL score and complication severity (Clavien-Dindo classification)

On day 3, the majority of patients experienced minor complications. Grade I complications were observed in 86.7% of patients (n = 78); Grade IIIA complications requiring intercostal drainage occurred in 10% (n = 9); and Grade IV complications requiring ICU admission occurred in 3.3% (n = 3). No Grade II, IIIB, or Grade V complications were observed on day 3.

Higher Clavien-Dindo grades were observed exclusively in patients with STUMBL scores ≥ 11, with Grade IV complications confined to patients with STUMBL scores ≥ 16. A statistically significant association was observed between STUMBL score categories and Clavien-Dindo complication severity on day 3 (chi-square test, χ² = 65.2, p < 0.001).

On day 30, 63 patients (70%) had no complications (Grade 0), while 26 patients (28.8%) experienced minor complications (Grade I). One patient (1.1%) died due to acute respiratory distress syndrome and was classified as Grade V; this patient had a STUMBL score in the 26-30 range at presentation. No other Grade III or IV complications were observed at day 30. Early and short-term complication severity across STUMBL score categories is summarized in Table 5.

**Table 5:** Early (day 3) and short-term (day 30) complication severity across STUMBL score categories using the Clavien-Dindo classification Grade 0 indicates no complications. Grade I indicates minor complications that require only analgesia and physiotherapy. Grade IIIA indicates intervention without general anesthesia. Grade IV indicates life-threatening complications requiring ICU care. Grade V suggests death. STUMBL: STUdy of the Management of BLunt chest wall trauma.

STUMBL score category	Day 3 Grade I	Day 3 Grade IIIA	Day 3 Grade IV	Day 30 Grade 0	Day 30 Grade I	Day 30 Grade V
0–10	77	4	0	61	15	0
11–15	1	2	0	2	6	0
16–20	0	1	1	0	2	0
21–25	0	1	0	0	1	0
26–30	0	0	1	0	0	1
≥31	0	1	1	0	2	0

STUMBL score and length of hospital stay

The mean hospital stay was 6.26 ± 4.12 days. Increasing STUMBL score categories were associated with progressively longer hospital stays. Patients with STUMBL scores ≤ 10 had a mean stay of 1.21 days, compared to 13 days in those with scores ≥ 31 (Table 6). A significant association was observed between increasing STUMBL score categories and hospital length of stay (p = 0.031).

**Table 6:** Hospital length of stay across STUMBL score categories * Estimated risk derived from the original STUMBL score description and not calculated from the present cohort. STUMBL: STUdy of the Management of BLunt chest wall trauma.

STUMBL score category	STUMBL-estimated risk of complications*	Mean hospital stay (days)	Patients, n
0–10	13%	1.21	81
11–15	29%	3.33	3
16–20	52%	8.0	2
21–25	70%	7.0	1
26–30	80%	5.0	1
≥31	88%	13.0	2

ICU admission and mortality

Three patients (3.3%, n = 3) required ICU admission. All ICU admissions occurred in patients with STUMBL scores ≥ 16. The highest ICU admission rate was observed in the highest score categories. There was one in-hospital death (1.1%, n = 1), which occurred on day 5 of admission in a patient with a STUMBL score of 29.

Hence, increasing STUMBL score categories were associated with greater healthcare resource utilization. ICU admission occurred exclusively in patients with STUMBL scores ≥ 16. In addition, the mean hospital length of stay increased progressively across higher STUMBL score categories, reflecting escalating resource requirements in patients with higher STUMBL scores. A consolidated summary of all inferential statistical analyses, including the statistical tests used, corresponding test statistics, and p-values, is provided in Table 7.

**Table 7:** Summary of statistical associations between STUMBL score categories and clinical outcomes Chi-square test was used unless expected cell counts were <5, in which case Fisher’s exact test was applied. No regression or correlation analyses were performed, as the study was designed to evaluate clinical risk stratification rather than prediction. STUMBL: STUdy of the Management of BLunt chest wall trauma.

Outcome variable	Comparison	Statistical test	Test statistic	p-value
Rib fractures	STUMBL score categories	Chi-square	χ² = 28.92	0.00002
Pneumothorax	STUMBL score categories	Chi-square	χ² = 39.27	0.0000002
Hemothorax	STUMBL score categories	Chi-square	χ² = 48.16	0.000000003
Pulmonary contusion	STUMBL score categories	Chi-square	χ² = 3.33	0.19
Atelectasis	STUMBL score categories	Fisher’s exact	-	1.00
Clavien-Dindo grade (day 3)	STUMBL score categories	Chi-square	χ² = 65.2	<0.001
Length of hospital stay	STUMBL score categories	Kruskal-Wallis	-	0.031

## Discussion

This prospective observational study demonstrates an association between increasing STUMBL score categories and adverse clinical outcomes in patients presenting with blunt chest trauma. Building on the observed graded escalation in complications, this study demonstrates that STUMBL score categories reflect not only the presence but also the severity of clinical complications, as assessed by the Clavien-Dindo classification, longer hospital stays, and a greater need for ICU admission. Importantly, escalation in complication severity was observed predominantly in patients with higher STUMBL score strata, despite many patients appearing physiologically stable at initial presentation.

STUMBL score as a risk stratification tool

The STUMBL score was developed to provide a pragmatic, bedside method of identifying patients at increased risk of complications following blunt chest trauma. Prior validation studies have shown that higher STUMBL scores are associated with increased likelihood of adverse outcomes, including respiratory complications and ICU admission [6-8]. Our findings are consistent with these reports and further reinforce the clinical utility of the STUMBL score for early risk stratification.

Unlike broader trauma scores such as the ISS or TRISS, which were designed to assess global injury burden, the STUMBL score focuses specifically on thoracic injury while incorporating physiological reserve and comorbid factors known to influence outcomes [3,4]. This targeted approach may explain its ability to identify patients at risk of deterioration even when initial vital signs appear reassuring.

Novelty: association with complication severity

A key and novel contribution of this study is the systematic evaluation of complication severity across STUMBL score categories using the Clavien-Dindo classification. While previous studies have primarily focused on binary outcomes such as the presence or absence of complications, ICU admission, or mortality, fewer investigations have examined how risk stratification scores relate to the graded severity of clinical deterioration.

Our findings demonstrate a clear trend toward escalating Clavien-Dindo grades with increasing STUMBL score categories. Minor complications (Grade I) predominated in patients with low STUMBL scores, whereas moderate (Grade IIIA) and severe (Grade IV) complications were confined to higher score strata. This graded relationship provides clinically meaningful information beyond simple outcome prediction and may help clinicians anticipate the level of intervention required during hospitalization.

The application of the Clavien-Dindo classification in trauma populations has gained increasing acceptance, as it offers a standardized and reproducible framework for grading complication severity based on therapeutic consequences rather than subjective assessment [15]. Integrating this classification with a validated risk stratification tool such as STUMBL enhances the interpretability and clinical relevance of outcome assessment. The temporal assessment of complication severity at both early and short-term time points further strengthens the clinical relevance of STUMBL-based risk stratification.

Resource utilization and length of hospital stay

Increasing STUMBL score categories were associated with progressively more extended hospital stays and higher ICU admission rates. Patients with low STUMBL scores generally had short, uncomplicated hospital courses, whereas those with higher scores experienced prolonged admissions and greater resource utilization. This finding aligns with prior reports linking STUMBL score escalation to an increased need for higher level care [16].

From a systems perspective, early identification of patients at risk for prolonged hospitalization or ICU admission may facilitate more efficient allocation of healthcare resources. In high-volume emergency departments, the STUMBL score may serve as an adjunct to clinical judgment when determining the appropriate level of monitoring and disposition.

Radiological findings and clinical correlation

Rib fractures and thoracic complications such as hemothorax and pneumothorax were more frequently observed in patients with higher STUMBL scores. This association is expected, as the number of rib fractures is a direct component of the STUMBL score. However, the clustering of more severe radiological findings within higher score categories supports the construct validity of the score and its ability to reflect underlying injury burden.

Selective use of CT imaging in this cohort reflects real-world practice, where advanced imaging is often reserved for patients with concerning clinical or radiological features. Despite limited CT utilization, clinically significant complications were effectively identified through a combination of bedside assessment, chest radiography, and eFAST, highlighting the practical applicability of the STUMBL score in routine emergency care.

Clinical implications

The findings of this study suggest that the STUMBL score may be helpful not only for identifying patients at risk of complications but also for anticipating the severity of those complications. Patients with higher STUMBL scores may benefit from closer monitoring, aggressive analgesia, early respiratory physiotherapy, and a lower threshold for ICU referral. Conversely, patients with low STUMBL scores and stable clinical parameters may be safely managed with less intensive resource utilization.

Importantly, the STUMBL score should be viewed as an adjunct rather than a replacement for clinical judgment. Its strength lies in its simplicity, reproducibility, and ability to provide early, structured risk stratification at the bedside.

Limitations

This study has several limitations. First, it was conducted at a single center, which may limit generalizability. Second, the number of patients in higher STUMBL score categories was relatively small, reflecting the skewed distribution of injury severity in blunt chest trauma. As a result, multivariable regression and correlation-based predictive modeling were not performed. The findings should therefore be interpreted in the context of clinical risk stratification, reflecting graded associations rather than causal inference or outcome prediction.

Third, the low incidence of certain complications, including pneumonia and mortality, limits the ability to draw definitive conclusions about these outcomes. Finally, selective use of CT imaging may have led to underdetection of minor pulmonary injuries; however, clinically significant outcomes were captured through longitudinal follow-up.

Given the selective imaging strategy and modest representation in higher STUMBL strata, the findings should be interpreted as exploratory associations within a real-world clinical context rather than definitive validation of graded severity discrimination.

Future directions

Future studies with larger, multicenter cohorts are needed to validate the association between STUMBL score categories and complication severity and to explore the incremental value of multivariable modeling. The integration of the STUMBL score into standardized trauma pathways and its impact on clinical decision-making and resource utilization represent important areas for further research.

## Conclusions

This prospective observational study demonstrates a clear and graded association between increasing STUMBL score categories and adverse clinical outcomes in patients with blunt chest trauma. Higher STUMBL score categories were associated with increased complication severity and healthcare utilization.

By correlating STUMBL score strata with standardized complication severity rather than binary outcomes alone, this study provides clinically meaningful insight into the escalation of risk following blunt chest trauma. These findings support the use of the STUMBL score as a practical, bedside clinical risk stratification tool to assist early clinical decision-making, guide monitoring intensity, and inform resource allocation in the acute care setting.
